# Workplace Violence in Outpatient Physician Clinics: A Systematic Review

**DOI:** 10.3390/ijerph17186587

**Published:** 2020-09-10

**Authors:** Lisa Pompeii, Elisa Benavides, Oana Pop, Yuliana Rojas, Robert Emery, George Delclos, Christine Markham, Abiodun Oluyomi, Karim Vellani, Ned Levine

**Affiliations:** 1 Department of Pediatrics, Baylor College of Medicine, Houston, TX 77030, USA; Elisa.Benavides@bcm.edu (E.B.); Abiodun.Oluyomi@bcm.edu (A.O.); 2 School of Public Health, University of Texas Health Science Center, Houston, TX 77030, USA; ospop2013@gmail.com (O.P.); robert.j.emery@uth.tmc.edu (R.E.); George.Delclos@uth.tmc.edu (G.D.); Christine.Markham@uth.tmc.edu (C.M.); 3 Department of Population Health, The University of Texas at Austin, Austin, TX 78712, USA; Yuliana.reds@gmail.com; 4 Threat Analysis Group, LLC, Houston, TX 77407, USA; kv@threatanalysis.com; 5 Ned Levine & Associates, Houston, TX 77025, USA; Ned@nedlevine.com

**Keywords:** violence, workplace violence, workplace aggression, primary care, outpatient physician clinic

## Abstract

Workplace violence (WPV) has been extensively studied in hospitals, yet little is known about WPV in outpatient physician clinics. These settings and work tasks may present different risk factors for WPV compared to hospitals, including the handling/exchange of cash, and being remotely located without security presence. We conducted a systematic literature review to describe what is currently known about WPV in outpatient physician clinics. Six literature databases were searched and reference lists from included articles published from 2000–2019. Thirteen quantitative and five qualitative manuscripts were included which all focused on patient/family-perpetrated violence in outpatient physician clinics. No studies examined other violence types (e.g., worker-on-worker; burglary). The overall prevalence of Type II violence ranged from 9.5% to 74.6%, with the most common form being verbal abuse (42.1–94.3%), followed by threat of assault (14.0–57.4%), bullying (2.5–5.7%), physical assault, (0.5–15.9%) and sexual harassment/assault (0.2–9.3%). Worker consequences included reduced work performance, anger, and depression. Most workers did not receive training on how to manage a violent patient. More work is needed to examine the prevalence and risk factors of WPV in outpatient physician clinics for purposes of informing prevention efforts in these settings.

## 1. Introduction

Workplace violence (WPV) including incivility, bullying, verbal abuse, physical threats, and physical assaults can result in serious adverse consequences for healthcare workers (HCWs) [1,2,3]. WPV has been extensively studied in hospitals and nursing homes [1,4,5,6]; yet little is known about WPV in outpatient physician clinics [7].

Outpatient physician clinics, which are part of the larger ambulatory care system, are responsible for providing primary care to patients in an outpatient, non-hospital setting [8]. Physician clinics play an important role in the health of our communities. Access to primary care has been positively associated with improved health outcomes [9], a reduction in hospital admissions [10,11], and a reduction in mortality [12]. Outpatient physician clinics typically employ physicians, physician assistants/nurse practitioners, nurses, nursing assistants, technicians, social workers, general operations managers, and administrative staff. A significant number (e.g., 221,000 U.S.) of healthcare workers are employed in these settings worldwide [13,14], and are responsible for managing more than one billion clinic visits annually [15,16].

Similarities exist between hospital and clinic workers with respect to their interactions with patients and patients’ family members, which could place them at risk for patient-perpetrated WPV (Type II violence). However, the type of care that clinic workers provide, their work tasks, and the environment where they work may make them more vulnerable to violence. For example, the handling and exchanging of cash is typically conducted in these clinics, and clinics can also be located in resource poor neighborhoods, both of which have been associated with crime-related violence (Type I violence) in other industries [17,18]. The small size of these establishments may also offer little protection to workers who experience worker-on-worker violence (Type III violence), or domestic violence that spills into the workplace (Type IV violence). To date, little has been reported on the WPV risk in outpatient physician clinics [7]; therefore, we conducted a systematic review to describe what is currently known, including violence prevalence, risk factors, prevention practices, reporting mechanisms, as well as worker consequences.

## 2. Materials and Methods

Using the preferred reporting items for systematic reviews and meta-analyses (PRISMA) guidelines [19], a systematic review was employed to collect data from published literature in English using electronic bibliographic databases from January 2000 to July 2019. Databases included were Medline, PubMed, CINAHL, Scopus, Google Scholar, and OpenGrey. The Medical Subject Headings (MeSH) terms and search strategy used were a combination of the following: (‘ambulatory care or ambulatory care facilities’ OR ‘outpatients’ OR ‘family practice or general practice’) AND (‘health facilities’); (‘violence’) OR (‘harassment, non-sexual, or sexual harassment or bullying) OR (‘aggression’) OR (‘crime’) OR (‘crime victims’) or (‘abuse.mp’) AND (‘workplace’) OR (‘workplace violence’) OR (‘occupational violence.mp’); (‘attitude of health personnel’) OR (‘health personnel’) OR (‘medical receptionist’). These strings of searches were further combined as search sets using Boolean operators (AND, OR). In addition, reference lists of the selected articles were used to identify and include additional relevant articles in our systematic review.

For this study, we included all violence types (Types I–IV), as well as all forms of WPV ranging from incivility, bullying, verbal abuse, physical threat, physical assault, and sexual assault [1,20]. Articles were excluded if: (1) the main exposure of interest was not WPV; (2) the study did not include workers in outpatient physician clinics or primary care physician clinics; (3) the study did not discern between different violence types (e.g., Type II versus Type III) in their analysis; (4) the study involved clinic workers but examined risk outside the clinic setting (e.g., patient’s home); or (5) the article was not original research (e.g., letter to the editor, editorial, conference reporting, or news article). No further exclusions were made with regard to study design or analytical approach. Two researchers independently screened all titles and abstracts, and later full text articles, in order to determine inclusion. When there were discrepancies between the two, a final decision was made by the study lead.

## 3. Results

**Figure 1:**
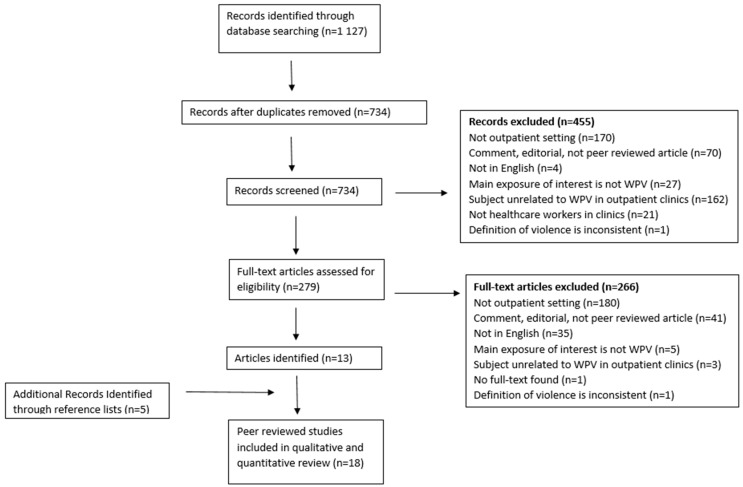
Preferred reporting items for systematic reviews and meta-analyses (PRISMA) overview of literature search and review of studies.

The systematic review initially identified 1127 articles which were reduced to 734 after duplicates were deleted (Figure 1). We screened 734 titles and abstracts from which 455 articles were excluded. A full text review was then conducted on 279 articles, with 266 that were excluded, and that 13 met the inclusion criteria. An additional five articles were identified through the articles’ reference lists resulting in 18 manuscripts included in our review (Table 1).

Of the 18 articles, 13 were quantitative studies and five were qualitative. All studies examined the prevalence of patient/family perpetrated violence (Type II) against HCWs, their experiences and potential risk factors, while none examined other violence types (Types I, III, or IV). These studies took place in various countries including Australia, Barbados, England, Ireland, Saudi Arabia, Serbia, Spain, and Turkey.

The 13 quantitative manuscripts were cross-sectional. Twelve used surveys to collect data on WPV experiences and outcomes, while one retrospectively analyzed reported Type II aggressions over a period of 24 months that HCWs reported into a clinic-based reporting system. Most of these studies (*n* = 10) asked HCWs to recall type II violent events in the previous 12 months, while two did not report the prevalence period. The response rates for studies ranged from 21% to 96%, with six studies having a response rate greater than 50%.

The qualitative manuscripts (*n* = 5) reported perceptions of WPV risk, experiences, and contributing factors. Three manuscripts focused specifically on physicians (general practitioners) and two on receptionists, who are front line workers that have initial contact with patients when they enter or call the clinic. Focus groups were the primary method of data collection with some qualitative questionnaires used to investigate the individual characteristics of the patient, physical environment of the consultation, physician vulnerability, psychological and emotional wellbeing, work satisfaction, and performance.

Workplace violence prevalence: All studies focused on Type II violence, while none focused on Types I, III, or IV. Among the 11 quantitative studies, the definition of workplace violence varied with most that examined physical assault (*n* = 12) and verbal abuse (*n* = 11), followed by physical threat (*n* = 8), sexual assault/harassment (*n* = 7), bullying (*n* = 2), and intimidation (*n* = 2). One study did not examine any specific forms of violence [21]. Among these 11 studies, the overall prevalence of Type II violence ranged from 9.5% to 74.6%. Verbal abuse was the most commonly reported form, with a prevalence ranging from 42.1% to 94.3%. The frequency with which some workers experienced WPV was emphasized by one participant in a qualitative study among receptionists [22] who stated: “*We get abused probably nearly every day, verbally, by different people and I think you can only take so much before you’re going to explode*.” Verbal abuse occurred both in-person and over the phone, especially directed at receptionists. In this same qualitative study [22] one participant stated, “*There’d always be the verbal violence over the phone, and I think that’s just part of the job. It’s probably not accepted but it does come with the job as receptionist. You get a lot of aggressive people over the phone*.” HCWs also experienced threat of assault with a prevalence range of 14% to 57.4%, 2.5% to 5.7% for bullying, 0.5% to 15.9% for physical assault, 0.2% to 9.3% for sexual assault and sexual harassment, and 22% for intimidation.

Worker characteristics: Eight studies reported the frequency of WPV stratified by worker (victim) characteristics (Table 2). Two studies by El-Gilany et al. [23] and Al-Turki et al. [24] reported male workers were more likely to experience some form of WPV compared to female workers whereas three studies reported females were more likely to experience WPV compared to males.

Among occupational groups, doctors reported a range in WPV prevalence from 24.4% to 59.3%, nurses 9.5% to 62.1%, receptionists 15.1% to 68.4%, and technicians 24.5% to 40%. With regard to clinic location, some studies found that clinics in rural locations had a higher WPV prevalence (22.0–85.1%) relative to those located in urban areas (21.4–57.7%). One study among receptionists highlighted that the 12-month prevalence of telephone verbal abuse by patients (60.0%) was on par with face-to-face verbal abuse (55.0%) (data not shown) [27].

With regard to years of work experience and WPV, the findings were mixed. El-Gilany et al. [23] reported an inverse relationship between violence and years of work experience, as HCWs with less than four years of work experience had a higher WPV prevalence (34.8%) compared to those who had work experience between 5 to 9 (25.8%), and 10 or more years of (23.9%). Conversely, Fisekovic et al. [28] observed a direct relationship between those who had fewer years of work experience and WPV, those with 10 or fewer years of experience had a lower prevalence of WPV (20.6%) relative to those with 11 to 20 years (33.5%) and more than 20 years (45.9%).

Three studies examined risk factors that contributed to Type II WPV (Table 3). HCWs who worked evening or night shifts (31.2–64.1%) were more likely to experience WPV compared with those working morning shifts (18.9–38.0%). Two studies reported the perpetrator was more likely to be the patient with a prevalence of 67.8% to 71.5% compared to a companion or relative of the patient (20.3% to 28.1%) [24,34]. In the study by El-Gilany et al. [23], the companion was more likely (68.1%) to be the perpetrator compared to the patient (23.1%). Al-Turki et al. [24] stated the perpetrator was both the patient and the companion in 3.3% of the reported events.

Perpetrator characteristics: With regard to perpetrator’s age, there was not a consistent pattern across studies. Al-Turki et al. [24] and Rincon et al. [34], reported the perpetrator was more likely to be older, with the highest WPV prevalence (31.7%) among perpetrators aged 46 years and older [24] and 60.4% in perpetrators aged 40 years old [34]. Conversely, El-Gilany et al. [23], reported the opposite with perpetrators who were aged 40 years and older (5.9%) relative to those younger than age 20 (25.0%).

Contributing and contextual factors: Three studies [23,24,34] ascertained factors that HCWs believed contributed to WPV which ranged from issues surrounding clinical care, clinic characteristics, security and patient factors. Contributing factors surrounding patient care included misunderstandings between HCW and the perpetrator (37.1–40.7%), unmet service needs (36.3–72.2%), and communication barriers (23.5%). Some clinic factors included overcrowding of the clinic (33.3–65.9%) and long wait times (17.0–32.5%), in addition to security factors, which included lack of security (39.4%), lack of protective measures (13.6%), and lack of penalty for the perpetrator when they were violent (49.6–67.2%). Contributing patient factors included their reaction to either an injury, accident, or illness (0.8–56.9%), their lack of knowledge about their health condition (30.1%), being mentally ill (20.9%), use of drugs (12.9%), and failure to receive a request for work leave or a sick note from the clinician (5.0%). The perception of violence was also studied in focus groups with physicians [35,36,37] in which they attributed some of the underlying causes to patients’ psychiatric disease, illicit drug use, sexual motivations, poverty and social dislocation. Some physicians stated that WPV had “*very much to do with the rise in the drug culture…that’s probably one big change that there’s an increase in drug use*” and “*…unfortunately an increase in the level of poverty that also breeds desperation and a feeling of [being] disadvantaged and angry with society*” [37].

Worker consequences and coping: Several studies examined aspects regarding workers’ emotional and physical consequences of the event. Among these, a few reported a high percentage (56.6–90.4%) of workers who indicated they did not experience any adverse consequences [24,34]. Among those that did, anxiety, fear, or helplessness (17.4–50.3%) was commonly reported, as well as reduced job satisfaction (69.2%), reduced work performance (30.1–31.1%), feeling sad (2.5%), depressed (28.1%), or angry (69.9%) [21,23,24]. In the most serious cases, HCWs needed psychological support (3.0–5.8%) [26,34].

Magin et al. [22] interviewed receptionists and reported that they took their fears and concerns home, with one participant who stated, “*It has kind of followed me home and I will think about it…I’ll just be thinking about it and I will [be] kind of upset. You just kind of replay it in your head and go ‘oh that was awful.*” In this same study, in-person abuse resulted in more distress and greater fear for escalation with a receptionist stating “*…it’s really nerve racking…It’s scarier, cause you think ‘I don’t know if they [violent patients] are coming back or are they waiting outside?’*” Bayman and Hussain [25] reported that receptionists who had been victims of WPV were approximately five times more likely to worry about being threatened or attacked again compared to those who had not been victims of WPV (data not shown).

When asked about coping with WPV events, almost half of the victims (40.7%) reported by El-Gilany et al. [23] indicated that they pretended the violent event did not happen. It was common for victims to report they did not take part in any form of coping mechanism (32.1–48.0%) [23,24] or they coped by themselves (53.3%) [24], while a small proportion took time off of work (5.3–7.0%) or sought counseling (3.0%) [26,34]. A substantial proportion did indicate that they confided in their colleagues (27.0–66.6%) [23,26] or their family or friends (13.8–39.4%) [23,24].

Reporting of WPV events: Only one study provided in-depth information about formal reporting of WPV events. Al-Turki et al. [24] indicated that among study respondents, only 30.9% reported the event to their superiors, and fewer (4.9%) called the police. In this same study, 58.8% of HCWs said there was a system at their workplace for reporting, while almost one-fourth (22.8%) did not know if one existed. Three additional studies mentioned some aspect of reporting with Chambers and Kelly [26] indicating the police were called for 16% of the events, El-Gilany et al. [23] indicated that among those that incurred an event only 1.7% reported it to a supervisor, while Fisekovic et al. [28] indicated that among those that incurred a WPV event, 48.6% were encouraged to report but did not indicate if they actually reported.

WPV prevention efforts: Various WPV prevention practices were mentioned across studies. Two studies examined the type of training HCWs had undergone pertaining to WPV prevention. In the study by Bayman and Hussain [25] 92% of respondents received training in the role of a physician’s receptionist, and 78% received training on how to deal with violent or difficult behavior from patients and their companions. Receptionists who did undergo training felt significantly safer at work, but there were still receptionists that worried about being threatened while at work (27%) or being physically attacked (22%) [25]. In the study by Chambers and Kelly [26], 28% of their practices surveyed had a policy in place for managing WPV and 13% of receptionists had received education that taught them how to deal with violence at work. In a small study of receptionists in the U.K. [27], verbal, threatening, and physical WPV were examined before and after the implementation of a national zero tolerance campaign in the U.K.; the researchers observed no changes in WPV events post-implementation.

A response seen from physicians as secondary measures to prevent violence involved changes in the built environment of the clinic to prevent verbal abuse from escalating to physical violence [35]. Physical boundaries were seen as important to provide safety to HCWs: “*We’ve got a very high counter that nobody can sort of like see behind.*” and “*[I]n our practice ah, we have a whole series of um, anti-violence measures. Um, the very first ah, every room has two doors*” [35]. Another physician indicated, *“… we have a duress alarm which rings police with a siren, and we have two methods of activating that at the front desk, and then we have a paging system in our rooms where we have a page button on our phones”* [35]. Magin et al. [38] interviewed receptionists who felt safe with the addition of higher counter tops, acrylic barrier between patient and receptionist, and lockdown systems in their clinics: “*I think it makes people feel more secure. Much, much more secure. [We] had people leaning over the desk shouting and spitting, you know, it was gross. [Perspex and lockdown] is where every general practice is going, it’s where every accident and emergency practice has already gone.*” and “*I like it. The other day, a lady came in and she became a bit agitated…She mumbled under her breath something about you know, ‘If we can’t get seen I’m going to come back and knock your block off.’ I just thought, ‘thank goodness for the glass.*” Receptionists felt safer from physical assault with a physical barrier between them and the clinic’s visitors: “*They can’t spit at you as much. Yeah they could still yell at you and all that kind of stuff, but they can’t throw a chair.*” However, some receptionists felt that this practice would be uninviting: “*Well I don’t think it would be very good coming in as a patient. I think you’d immediately sort of feel, ‘this can’t be a very safe place to be, otherwise people wouldn’t be behind those big Perspex walls’…I think it would be off-putting for them.*”

Removing patients from the clinic was also a common theme in a focus group conducted by Magin et al. [35]. These patients were banned from the clinics due to concerned physicians based on a patient’s violent, aggressive, or drug-seeking behavior. One doctor reported, “*We also have a list of people who under no circumstance will they be given appointments and no appointments will be given to their relatives.*”

## 4. Discussion

To date, only a small number of manuscripts pertaining to WPV in outpatient physician clinics have been published over the twenty-year period of this review. The findings from several of the studies in our review indicate that a high proportion of workers experienced patient/visitor-perpetrated verbal abuse, while not an insignificant proportion experienced physical threats and physical assaults, which were similar to estimates reported by hospital workers in prior studies [1,2]. While these findings are significant given the large number of workers employed in these settings worldwide, they are based largely on studies that used convenience sampling among populations not representative of healthcare workers. To provide reliable and generalizable WPV prevalence estimates in the outpatient physician setting, larger probability samples are needed [39,40].

All studies focused on patient/visitor-perpetrated (Type II) violence, while none focused on Types I, III, or IV. Among the 11 quantitative studies, the definition of WPV varied with most that examined physical assault (*n* = 12) and verbal abuse (*n* = 11), followed by physical threat (*n* = 8), and sexual assault/harassment (*n* = 7), bullying (*n* = 2), and intimidation (*n* = 2). The variations in definitions limited our ability to compare findings across studies. None of the studies examined the other types. The paucity of studies examining these other violence types should not be used to infer that these workers are not at risk for criminal-type violence (e.g., burglary) or bullying from coworkers. A large body of literature outside of this setting has highlighted worker-on-worker (Type III) violence experienced by healthcare workers [3]. Further, findings from the 2019 International Association for Healthcare Security and Safety’s (IAHSS) annual Healthcare Crime Survey [41] indicated that hospital workers experienced serious crime-related events such as robbery, burglary, and theft (Type I), as well as domestic or personal relationship-related violence that occurred in the workplace (Type IV). Howard and others [42,43] have emphasized the importance of examining all violence types for informing prevention strategies given that differences in the perpetrator’s profile and motives, and workplace and workers affected can vary by violence type. These details are needed to inform targeted prevention efforts.

Physician clinics are located in various settings, including high-density commercial developments (e.g., central business and/or shopping districts), as well as resource limited areas, which have been positively associated with clusters of crime [44,45]. Only three studies [23,31,33] in our review examined clinic location using a crude measurement of rural versus non-rural, and the findings were mixed. A more in-depth examination of the geospatial relationship between WPV in outpatient clinics and their surrounding characteristics of population, traffic, and businesses would be informative and inform prevention efforts.

Only a few studies examined factors that workers attributed to patient-perpetrated violence. Of those, numerous factors were described including, patient drug seeking, mental illness, alcohol abuse, dissatisfaction with care, limited access to care, being in pain, and long wait times [20,22,35]. While these three studies provide some insight into the factors that may place workers at risk, the measurement of factors was inconsistent across studies, and only two [23,34] performed more advanced multivariate modeling for purposes of assessing their contribution to the risk of violence. Further, several of these factors are considered modifiable or manageable risk factors through targeted interventions such as worker de-escalation training [46], yet only one study [25] examined this type of intervention.

Some of the identified WPV risk factors are similar to those identified in hospital-based studies [2]; however, these events occur in a very different work environment, which may require different prevention measures. For example, some studies pertaining to receptionists focused on the environmental protection through the use of acrylic barriers at the front desk, as well as clinic lock-down procedures—two types of prevention measures atypical to the inpatient hospital unit setting. While these environmental barriers made receptionists feel safer and prevented them from being spit on or assaulted by patients, little is known about how pervasively these are used and their overall effectiveness. Another prevention effort specific to the outpatient setting was the removal of patients from the practice that had a known history violent behavior [35]. In a study outside our review, Pickin et al. [47] found that the primary reason general practitioners removed patients from their practice was violent, threatening or abusive behavior (59%), followed by drug seeking and drug misuse (12%). Overall, our review suggests a lack of broader prevention guidelines and efforts, which may explain why some workers were left to take matters in to their own hands by learning self-defense, or carrying a weapon while at work [35].

The reporting of WPV by workers is essential to the management and prevention of these events. The under-reporting of WPV by workers is not new [48], and the lack of information about reporting in this review suggests its relevance has continued to be overlooked. Clinics and clinic systems should have a system for capturing WPV events in which workers are trained on where and when to report [49,50]. These data are essential not only for identifying risk factors, but for assessing the effectiveness of prevention efforts.

The findings from this review were limited due to methodological weaknesses in this body of literature. The majority of the studies were descriptive in nature, many with small sample sizes, a few with low response rates [29,44], with few [23,28] that used robust analytical methods to examine WPV risk factors. The variation in how WPV was defined across studies limited comparisons across studies, as well as differences in their data analysis and presentation of data findings. A strength, however, was the collection of qualitative studies that complemented and provided context to the quantitative findings, as well as giving workers a voice about their experiences with workplace violence.

## 5. Conclusions

Workplace violence is recognized as a significant public health issue. Findings from this review suggest that outpatient clinic workers are at high risk for experiencing violence from patients or clinic visitors, and that they experience serious emotional consequences of depression, chronic fatigue, poor job satisfaction, and feeling ashamed. This review also highlights numerous gaps and limitations that warrant further research. Data regarding WPV events need to be captured from workers for purposes of describing the full scope of the problem, and to inform prevention efforts. This includes examining all types of violence beyond Type II violence. Larger longitudinal studies are needed that use rigorous analytical techniques that would allow for inferences of causal associations between WPV and potentially modifiable risk factors, as well as employee outcomes. Such findings can lead to new interventions to eliminate or mitigate risk factors and highlight employee outcomes to assess during intervention effectiveness studies. For example, it would be informative to examine the effectiveness of WPV prevention interventions, such as de-escalation training, that has shown promise in other types of healthcare settings. Workers in outpatient physician clinics provide essential primary care services to our communities and greater attention should be given to their safety and well-being while at work.

## Figures and Tables

**Table 1:** Research articles pertaining to workplace violence in outpatient physician clinics (*n* = 18).

Author, Year, Country	Study Design	Sample Size	Work Group(s)	Data Collection Methods	Response Rate	Prevalence Period	Violence Prevalence							
							Violence Type	All Types	Verbal Abuse	Threat of Assault	Bully-ing	Physical Assault	Sexual Assault/Harass	Intimi-Date
Ayranci et al., 2006, Turkey [21]	CS	151	All	Survey	NA ^a^	12 m	Type II	49.0% ^b^	-	-	-	-	-	-
Magin et al., 2009, Australia ^g^ [22]	Q	19	RT	Interviews/QS	55.0%	-	Type II	-	-	-	-	-	-	-
El-Gilany et al., 2010, Saudi Arabia [23]	CS	1091	All	Survey	96.1%	12 m	Type II	27.7%	52.4%	33.3%	2.5%	5.0%	NR/1.4%	-
Al-Turki et al., 2016, Saudi Arabia [24]	CS	270	All	Survey	NR	12 m	Type II	45.6%	94.3%	-	-	6.5%	-	22.0%
Bayman and Hussain 2007, England [25]	CS	207	RT	Survey	68.0%	12 m	Type II	-	-	26.0%	-	0.5%	-	-
Chambers and Kelly 2006, Ireland [26]	CS	271	RT	Survey	68.0%	NR	Type II	62.0%	99.0%	31.0%	-	6.0%	-	-
Dixon et al., 2003, England [27]	CS	171 ^c^	RT	Survey	78.0%	12 m	Type II	-	68.0%	14.0%	-	4.0%	<5.0%	-
Fisekovic et al., 2015, Serbia [28]	CS	1526	All	Survey	86.9%	NR	Type II	52.6%	43.5%	-	5.7%	1.9%	NR/0.4%	-
Forrest et al., 2011 Australia [29]	CS	804	MD	Survey	26.3%	12 m	Type II	-	58.0%	-	-	6.0%	0.1/6.0%	-
Gascon et al., 2009, Spain [30]	CS	440	MD, RT, AD	Survey	NA ^a^	12 m	Type II	-	59.3% ^d^	57.4% ^d^	-	15.9% ^d^	-	-
Koritsas et al., 2007, Australia [31]	CS	211	MD	Survey	21.1%	12 m	Type II	57.0%	44.0%	-	-	3.0%	1.0/8.0%	22.0%
Magin et al., 2005, Australia ^e^ [32]	CS	528	MD	Survey	49.0%	12 m	Type II	63.7%	42.1%	23.1%	-	2.7%	0.2/9.3%	-
Magin et al., 2011, Australia [33]	CS	125	All	Survey	55.0%	12 m	Type II	59.3% (MD); 74.6% (other)	64.0%	24.8%	-	1.0%	NR/8%	-
Rincon-del Toro et al., 2016, Spain [34]	CS	11,525	All	System Reported Events	NA	24 m	Type II	9.5%	-	-	-	-	-	-
Magin et al., 2006, Australia ^e^ [35]	Q	18 FG/ 154 QS ^f^	MD	FG/QS	-	-	Type II	-	-	-	-	-	-	-
Magin et al., 2008, Australia ^e^ (a) [36]	Q	18 FG/ 154 QS ^f^	MD	FG/QS	-	-	Type II	-	-	-	-	-	-	-
Magin et al., 2008, Australia ^e^ (b) [37]	Q	18 FG/ 154 QS ^f^	MD	FG/QS	-	Over career	Type II	75.0%	-	-	-	-	-	-
Magin et al., 2010, Australia ^g^ [38]	Q	19	RT	Interviews	-	-	Type II	-	-	-	-	-	-	-

Study Design: CS = cross sectional; Q = Qualitative. Sample size: FG = focus groups; QS = qualitative surveys. Work groups: MD = physician; AD = administration; RT = receptionist; all = all clinic and nonclinical staff; response rate: m = month; NA = Not applicable; NR = Not reported. ^a^ Study examined hospital/clinic locations and response rate for outpatient/primary care clinics not provided; ^b^ examined all health care settings and provides only overall violence prevalence for outpatient clinics; ^c^ calculated sample size from percentages given; ^d^ re-calculated rates to include total for rural and urban primary care clinics; ^e^ manuscripts pertain to the same study; ^f^ 18 participants from focus groups and 154 from qualitative questionnaires; ^g^ manuscripts pertain to the same study.

**Table 2:** Prevalence estimates of outpatient physician clinic WPV by worker and clinic characteristics.

	Al-Turki (*n* = 270) [24]	Chambers (*n* = 271) [26]	Dixon (*n* = 171) [27]	El-Gilany (*n* = 1091) [23]	Fisekovic (*n* = 1526) [28]	Forrest (*n* = 804) [29]	Koritsas (*n* = 211) [31]	Magin, 2005 (*n* = 528) [32]	Magin, 2011 (*n* = 125) ^e^ [33]	Rincon-del Toro (11,525) [34]
**Sex**										
Male	48.2%			28.6%	15.2%			54.0%/25.0% ^d^	53.3%	
Female	44.0%			26.2%	84.8%			46.0%/75.0% ^d^	72.6%	
**Occupation**										
Doctor	44.4%			41.2%	28.6%	26.3% ^b^	57.0%	63.7%	59.3%	24.4%
Nurse	36.0%			24.7% ^a^	62.1%					9.5%
Receptionist	68.4%	62.0%	68.0% ^c^							15.1%
Technician	40.0%			24.5%						
Other/unknown	71.4%			37.1%	9.2%					3.3
**Clinic Location**										
Urban				21.4%			43.0% ^c^		57.7%	
Rural				22.0%			51.0% ^c^		85.1%	
**Work experience**										
≤10 years (1 to <5; 5 to <10)				(34.8%; 5.8%)	20.6%					
11–20 (10+)				(23.9%)	33.5%					
>20					45.9%					

^a^ Includes midwives; ^b^ excludes property damage/theft; ^c^ verbal abuse only; ^d^ estimates of low-level violence (verbal abuse, property damage/theft, threats, slander)/high level violence (physical abuse, sexual abuse, stalking, sexual harassment); ^e^ estimates for other individual work groups not reported.

**Table 3:** Factors contributing to workplace violence events in outpatient physician clinics.

WPV Event Factors	Al-Turki (*n* = 123) [24]	El-Gilany (*n* = 913)/(*n* = 302) ^a^ [23]	Rincon-del Toro (*n* = 1157) [34]
**Time of Event**			
Morning shift	38.0%	18.9%	
Evening/night shift	64.1%	49.8/31.2%	
**Place of Event**			
Inside workplace	99.2%		
Consultation			62.7%
Reception counter			0.3%
**Identity of Perpetrator**			
Patient	71.5%	23.1%	67.8%
Companion	20.3%	68.1%	28.1%
Patient and Companion	3.3%		
**Sex of Perpetrator**			
Male	65.9%	95%	56.8%
Female	30.1%	5.0%	43.2%
Both	4.1%		
**Age of Perpetrator**			
<20	7.3%	25.0%	
20–39 (19–30; 31–40)		59.9%	(12.9%; 26.7%)
21–45 (40+)	61.0%	(5.9%)	(60.4)
≥46	31.7%		
**Workers’ Perceptions of Contributing Factors**			
**Patient Care/Communication Issues**			
Misunderstandings between HCW and perpetrator	40.7%	37.1% ^a^	
Unmet service or needs	36.6%	72.2% ^a^	36.3%
Language or communication barriers		23.5% ^a^	
**Clinic Factors**			
Overcrowding	33.3%	65.9% ^a^	
Long waiting times	32.5%		17.0%
**Security Factors**			
Lack of security in the clinic		39.4% ^a^	
Lack of protective measures		13.6% ^a^	
Lack of penalty for perpetrator	49.6%	67.2% ^a^	
**Patient Factors**			
Reaction to injury/accident/illness	0.8%	56.9% ^a^	
Lack of knowledge/illiteracy		30.1% ^a^	
Impatience (patient in a hurry)		58.9% ^a^	
Mentally ill		20.9% ^a^	
Drug abuse			12.9%
Request of work leave/sick note			5.0%

^a^ Sample size for contributors to violence (*n* = 302).
